# Evaluating Classification Consistency of Oral Lesion Images for Use in an Image Classification Teaching Tool

**DOI:** 10.3390/dj9080094

**Published:** 2021-08-12

**Authors:** Yuxin Shen, Minn N. Yoon, Silvia Ortiz, Reid Friesen, Hollis Lai

**Affiliations:** Department of Dentistry, Faculty of Medicine and Dentistry, University of Alberta, Edmonton, AB T6G 1C9, Canada; shen3@ualberta.ca (Y.S.); minn.yoon@ualberta.ca (M.N.Y.); ssortiz@ualberta.ca (S.O.); rtfriese@ualberta.ca (R.F.)

**Keywords:** dental education, dental hygiene education, educational technology, classification consistency, oral lesion

## Abstract

A web-based image classification tool (DiLearn) was developed to facilitate active learning in the oral health profession. Students engage with oral lesion images using swipe gestures to classify each image into pre-determined categories (e.g., left for refer and right for no intervention). To assemble the training modules and to provide feedback to students, DiLearn requires each oral lesion image to be classified, with various features displayed in the image. The collection of accurate meta-information is a crucial step for enabling the self-directed active learning approach taken in DiLearn. The purpose of this study is to evaluate the classification consistency of features in oral lesion images by experts and students for use in the learning tool. Twenty oral lesion images from DiLearn’s image bank were classified by three oral lesion experts and two senior dental hygiene students using the same rubric containing eight features. Classification agreement among and between raters were evaluated using Fleiss’ and Cohen’s Kappa. Classification agreement among the three experts ranged from identical (Fleiss’ Kappa = 1) for “clinical action”, to slight agreement for “border regularity” (Fleiss’ Kappa = 0.136), with the majority of categories having fair to moderate agreement (Fleiss’ Kappa = 0.332–0.545). Inclusion of the two student raters with the experts yielded fair to moderate overall classification agreement (Fleiss’ Kappa = 0.224–0.554), with the exception of “morphology”. The feature of clinical action could be accurately classified, while other anatomical features indirectly related to diagnosis had a lower classification consistency. The findings suggest that one oral lesion expert or two student raters can provide fairly consistent meta-information for selected categories of features implicated in the creation of image classification tasks in DiLearn.

## 1. Introduction

Exposure to oral lesion images is an important component of dental education and oral pathology education. In traditional classroom settings, lecturers show an image of oral lesions and discuss the feature of an image iteratively. The process of interpreting a diagnostic image is generally divided into three phases [1], namely: (1) psycho-physical, which identifies the appropriate examination technique and human anatomical system; (2) psychological, which provides a higher cognitive process in organizing the images into precepts; and (3) nosological, which integrates the medical knowledge required to produce a diagnosis.

To effectively train students to produce a diagnosis based on diagnostic images, students are required to process the image, incorporating an array of information such as the location, size, opacity, medical history, demographic information, and underlying histology [2]. While students are often given longitudinal case information, this inductive approach trains students through a step-by-step manner, drawing on their nuanced understanding of clinical information, features of the image, and a cognitive understanding to interpret the image [3]. Students beginning in the profession often do not possess the aptitude to follow the process required for integrating all of the necessary information. Rather than focusing on interpreting entire cases at the outset, our study will focus on providing students with opportunities to familiarize themselves with the definitions, signs, and features associated with various abnormalities; features that are required to interpret images in the nosological phase [3].

Studies have demonstrated varying effects in diagnostic training in image-based areas such as radiography. Specifically, students who received training on pathophysiological information had a better diagnostic accuracy than students who were trained using feature lists [4]. The preference of a pathophysiological primer suggests novice learners need to be exposed to the features and anatomical cues. This process is supported by the development of domain expertise knowledge [5], where expertise is developed through exposure to domain related categories, cues, and knowledge that are related to the presented visual features.

Incorporating computer-assisted instruction (CAI) to complement other forms of traditional instruction is advantageous as it allows students to learn independently and have the learning reinforced with immediate feedback. Using CAI has been shown to be equivalent in cognitive performance and superior in clinical performance compared with traditional didactic instruction [6]. Moreover, the students required comparable amounts of learning time but progressed on an individual basis while receiving immediate feedback to reinforce their learning. Research has also found that a blended learning approach (teaching method that uses asynchronous learning to complement synchronous learning events) to teaching radiology led to superior student performance when compared with traditional approaches [2].

### Overview of DiLearn

Spaced education is a pedagogical approach that focuses on the repetition of tasks that are separated or spaced through time to leverage the spacing effect in learning [7,8,9]. Students who are instructed in this method have yielded longer retention [7], and the immediate feedback provided electronically has shown to improve self-assessment [8].

To emulate this learning approach in the electronic environment, DiLearn organizes learning by modules. Each module contains a collection of images and is designed to train students on a defined topic through three stages, namely: learning, training, and assessment. In the learning phase—students are presented with images and are required to determine what clinical action is required (e.g., intervene by referring to a specialist). Students receive immediate feedback after an action (outline the type of feedback: correct, incorrect, information related to the features of the image). In the training phase, students undergo the same classification task with different images, with the only difference being that feedback is presented at the end of the exercise (e.g., total number correct) with a chance to review selections. Students have unlimited attempts at modules, and the modules are assembled randomly from a bank of images. After students have achieved a certain level of correct classification in the training phase, they are then allowed to attempt the assessment phase, where they complete the same classification task with a novel set of images and without immediate feedback.

**Figure 1:**
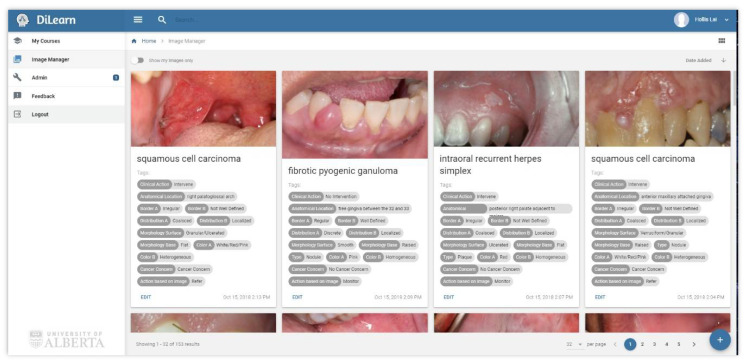
An image bank with its corresponding tags.

To create the learning modules, a large collection of images related to different presentations and diagnoses of oral lesions were needed. Each image then required meta-information, or tags based on a taxonomy of clinical features. Figure 1 illustrates examples of tags associated with images. The information provided in the tags can be used by instructors to define the classification students will choose in each module, or to balance the presentation of a specific feature presented in each module through searching and filtering (e.g., images of lesions on tongues; red lesions).

**Figure 2:**
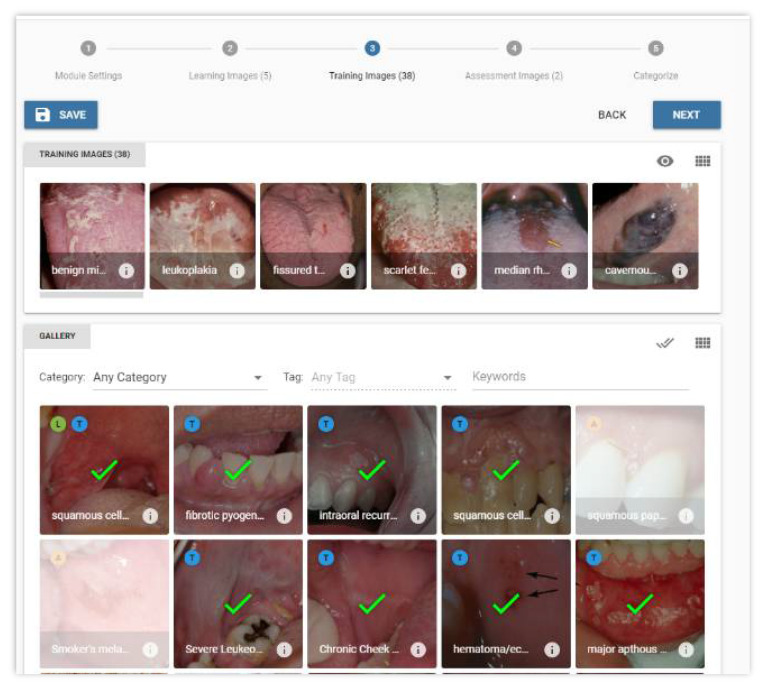
The selected images assigned to a stage.

**Figure 3:**
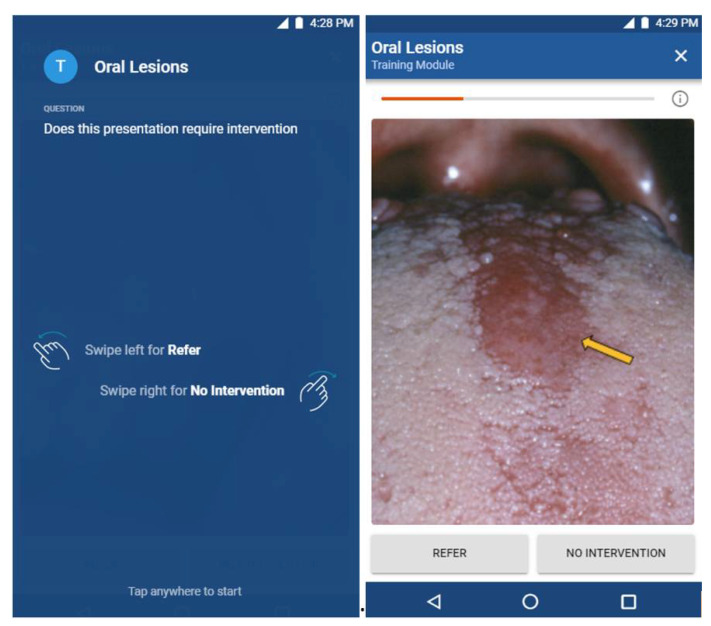
Screen captures of the student interface.

After a module is assembled, it can be administered to students to complete. Figure 2 illustrates how images can be assigned to a stage. The application requires university authentication to verify student identity. Figure 3 presents the smartphone interface for how the learning modules can be completed by students. Students will be presented with an oral lesion image; they are given the choice of either swiping left to choose the action of “refer” or swiping right to choose the action of “no intervention”. Given the importance of the meta-information and tags associated with each image, which are needed to develop learning modules and for providing the suggested action when presented with an oral lesion image in the student interface, the creation and validation of this meta-information structure is the focus of this study.

## 2. Materials and Methods

This study was approved at the University of Alberta by the Research Ethics Board (Pro00066469). To develop and evaluate a taxonomy for teaching oral lesions, the study was separated into two phases. In the first phase, a taxonomy of oral lesion descriptions was created. Two content experts were tasked to provide a list of features required to describe oral lesions in order to help students learn about oral pathology. Each expert was asked to inductively provide the features they commonly identify and distinguish in order to diagnose the presentation of an oral lesion. After the two experts provided their individual lists, they compared the two list of categories and features, and agreed on a single taxonomy of clinical features that could be used to describe an oral lesion, and used as tags for classifying the images. A glossary of terms was then developed that operationalized each feature using descriptive characteristics.

In the second phase, five participants were recruited for the study—three oral pathology specialists with expertise in oral cancer lesions and two senior dental hygiene students with training in oral lesions. A set of 20 oral lesion images was selected from DiLearn’s image bank. The five raters classified the 20 oral lesion images using the taxonomy developed in phase one. Responses were recorded and compiled into Microsoft Excel, after which the coded data were imported into IBM SPSS Version 23. Three levels of statistical analyses were carried out to evaluate the classification agreement among the three oral lesion experts, between the student raters, and among all five raters. Fleiss’ Kappa of the three oral lesion experts was computed to determine the overall agreement among the experts. The Cohen’s Kappa between the two student raters was used to determine the classification agreement between the student raters. With the inclusion of the student raters, an overall Fleiss’ Kappa of the five raters was computed to evaluate how the incorporation of the student raters contributed to the overall classification agreement. Cohen’s and Fleiss’ Kappa were chosen because of the categorical nature of the tags, and were interpreted based on Landis’ and Koch’s guidelines [10].

## 3. Results

In the first phase, two of the three content experts described and agreed on eight features for describing oral lesions. The eight features included clinical action, anatomical location, border, configuration, color, type, morphology, and localization. Each category then contained different definitions that described the morphological features of the lesion in the image. An example of the definitions for each category described in the taxonomy is provided in Table 1.

In the second phase, all five raters tagged a collection of 20 images from DiLearn’s image bank using the eight features provided in phase one. The category of the highest importance was clinical action, as this category provided the suggested intervention in the DiLearn’s student interface. Fleiss’ Kappa on clinical action among all three experts was calculated to be 1, showing perfect classification consistency among all three experts. Consistency between the two students for intervention was measured at 0.33 using Cohen’s Kappa, demonstrating a fair agreement between the students. Fleiss’ Kappa on clinical action decreased from 1 to 0.52 with the addition of the two student raters. Even with the decrease in Fleiss’ Kappa, there was still moderate agreement for clinical action after the inclusion of the two student raters.

Overall, classification consistency among the three experts ranged from perfect for clinical action to slight agreement for border regularity, with the majority of categories having moderate to fair agreement, as seen in Table 2. There was moderate agreement among the three experts for the anatomical location, color, and morphology, with Fleiss’ Kappa calculated to be 0.46, 0.55, and 0.54, respectively. Fleiss’ Kappa showed fair agreement for border definition, configuration, type, and localization.

The agreement between the two students ranged from substantial to poor. There was substantial agreement between the two students for color, as demonstrated by a Cohen’s Kappa of 0.70. Moderate to fair agreements between student raters were measured for the categories of clinical action, border regularity, anatomical location, configuration, type, and localization. A poor agreement was measured for morphology.

The overall Fleiss’ Kappa was calculated to evaluate the effect the student raters had on the classification consistency and to compare the student raters with the expert raters. After the inclusion of student raters, there was moderate to fair agreement for all of the categories, except for morphology. For the categories of border regularity, anatomical location, configuration, color, and type, the addition of the two student raters increased the Fleiss’ Kappa, suggesting an increase in agreement with the addition of the two less experienced raters. For the categories clinical action, border definition, morphology, and localization, the addition of the two student raters decreased the overall agreement, as demonstrated by a decrease in Fleiss’ Kappa.

## 4. Discussion

Oral cancer screening is often a routine part of the dental examination of patient, especially when examining those with risk factors for oral cancer. An oral cancer screening program in Taiwan targeting high-risk individuals demonstrated the effectiveness of reducing later stage oral cancers and oral cancer mortality [11]. The effectiveness of the oral visual screening in reducing mortality in high-risk individuals was corroborated by a randomized control trial in India [12]. A study by Hassona et al. showed that dental students’ ability to recognize oral lesions of concern was significantly correlated with oral cancer knowledge and knowledge about potentially malignant oral disorders [13]. To improve students’ future diagnostic ability and their ability to complete oral cancer screening, there is a need for increasing students’ exposure to oral lesions and for improving the educational methods used to deliver the content on oral cancer and oral lesions.

Tools and technology for active learning in health profession education are currently in high demand. In Saxena et al.’s article from 2009, crossword puzzles were introduced to the undergraduate medical curriculum to help students review and reinforce concepts and vocabulary learned in a pathology class [14]. Specially constructed content-relevant digital games were constructed by Kanthan et al., and helped improve academic performance in undergraduate pathology courses [15]. However, few tools have been created specifically for undergraduate dental and dental hygiene curriculums to learn oral pathology and oral medicine. There are even fewer tools that have been developed based on learning theories, and these tools are seldom evaluated. By introducing a new tool for image classification such as DiLearn, faculties from dentistry and dental hygiene can integrate active learning into oral pathology, which is traditionally delivered in a didactic format. The development of this learning tool also has the potential to integrate learning for students in other disciplines. Student performance data collected in the coming year should allow for an analysis of student learning to illustrate the learning curve of a given classification for diagnosis, a challenge that has not yet been done, despite the abundant research conducted on competency education in the health profession. Such learning curve information can be used to determine the appropriate amount of instruction and exposure required to master a specific oral pathology.

The validation evidence demonstrated in this study shows how the three experts can provide relatively consistent meta-information for the tagging of the 20 images as reflected by the fair to perfect agreement for all categories, with the exception of border regularity. The three experts reached consensus when categorizing clinical action, which is one of the more important categories in DiLearn, as this category provides the suggested action in the DiLearn’s student interface and will also be used to track student progress. The lower Fleiss’ Kappa for some categories can be explained partly by the lack of a definitive key or descriptors for each taxonomical category. For instance, when describing border, two of the expert raters chose one descriptor from “regular”, “irregular”, “well defined”, and “not well defined” to describe the border of the pathological lesions, while the other expert chose one descriptor from “regular” and “irregular” and one descriptor from “well defined” and “not well defined” to describe the border of the pathological lesions. It is suggested that the introduction of a multiple-choice format with set descriptors for each category be implemented for future classification tasks, as this strategy will likely increase the overall agreement between and among raters.

The agreement between the two student raters ranged from substantial to poor, with the majority of categories having moderate to fair agreement. The two students had the most agreement when classifying anatomical location and color, and the least agreement when classifying morphology, perhaps reflecting that the student raters were more comfortable when classifying categories such as anatomical location and color than morphology. The negative Cohen’s Kappa for morphology reflects that the students were not beating chance when it came to categorizing morphology. A negative Kappa value for morphology may suggest that students were less experienced with the characterization of morphology, or that the two student raters may have a different interpretation of the tags used for morphology.

Lastly, the effect the two student raters had on classification agreement was examined by comparing the Fleiss’ Kappa for the three experts with the Fleiss’ Kappa after the inclusion of the student raters. The inclusion of the two student raters increased the Fleiss’ Kappa for border regularity, anatomical location, configuration, color, and type. For the categories clinical action, border definition, morphology, and localization, the addition of the two student raters decreased the overall classification agreement. Though it has to be noted that clinical action had an initial Fleiss’ Kappa of 1, and the 0.001 decrease in Fleiss’ Kappa for localization was likely of negligible significance. With the exception of morphology, the inclusion of the two student raters did not have a negative impact on the overall classification agreement. On the contrary, the overall classification agreement increased in some cases when more raters were involved in the classification task.

One of the most important categories for DiLearn is the category of clinical action, as the tags associated with clinical action will be used in the student interface (Figure 3). In the student interface, students are presented with an oral lesion image and are given the choice to swipe left for “refer” or swipe right for “no intervention”. The three oral lesion experts had a Fleiss’ Kappa of 1 for the category of clinical action (the three oral lesion experts agreed on the clinical action when presented with an oral lesion image), suggesting only one oral lesion expert is needed for tagging the category of clinical action. With the inclusion of the two student raters, the overall Fleiss’ Kappa reduced to 0.523 for the category of clinical action, suggesting that the two student raters can still provide fairly consistent meta-information for tagging the category of clinical action. Given the time and effort required for subject matter experts to provide tags and to assemble the learning modules, and the relative agreement when more raters participate in the tagging task, a future study should investigate whether increasing the number of students in tagging may provide meta-information with similar precision when compared with experts.

## 5. Conclusions

With an increasing need to provide students with exposure to various presentations of oral pathologies, learning tools like DiLearn have the potential to facilitate an independent learning process. It must be noted, however, that the collection of accurate meta-information is a crucial step for enabling the self-directed active learning approach taken in DiLearn. The learning process of each module and the feedback provided to the students are reliant on accurate diagnostic outcomes. Validation evidence presented in this study suggests one oral lesion expert or two student raters can provide fairly consistent meta-information for select category of features implicated in the creation of the classification tasks, especially for the category of clinical action. The inclusion of the two student raters did not significantly alter the overall classification agreement, but future investigations should be carried out to investigate whether increasing the number of students in tagging may provide meta-information with similar precision as for the experts.

## Figures and Tables

**Table 1:** A description of the taxonomy and the keys used to code each image.

Category	Description
Clinical Action	intervene (1), or monitor or no intervention (0)
Border Regularity	regular (1) or irregular (2)
Border Definition	defined (3) or ill-defined (4)
Anatomical Location	tongue (1), gingiva (2), mucosa (3), palate (4), floor of mouth (5), lip (6), interdental papilla (7), vestibule (8), or alveolar bone (9)
Configuration	discrete (1), coalesced (2) multiple (3), or no applicable terms (4)
Color	white (1), yellow (2), pink (3), or red (4)
Type	exophytic (1), plaque (2), submucosal mass (3), macule (4), or ulcer (5)
Morphology	granular (1), smooth (2), ulcerated (3), verruciform (4), fissured (5), or lobulated (6)
Localization	localized (1) or non-localized (2)

**Table 2:** Fleiss’ and Cohen’s Kappa evaluating the classification agreement of 20 oral lesion images by three experts and two students.

	Fleiss’ Kappa		Cohen’s Kappa
	Experts	Overall	Between Two Students
Clinical Action	1	0.523	0.327
Border Regularity	0.136	0.224	0.348
Border Definition	0.347	0.256	0.097
Anatomical Location	0.464	0.539	0.571
Configuration	0.332	0.368	0.568
Color	0.545	0.554	0.695
Type	0.392	0.441	0.490
Morphology	0.537	0.146	−0.019
Localization	0.392	0.391	0.211

## Data Availability

The data presented in this study are available on request from the corresponding author.
